# The Surgical Treatment of Encephalocele

**Published:** 1891-01-24

**Authors:** 


### SURGERY.

### THE SURGICAL TREATMENT OF ENCEPHALOCELE.

This subject forms an entirely new chapter in the second edition that has just appeared of Dr. Ernest von Bergmann's work on the surgical treatment of diseases of the brain. He divides Encephaloceles into the two great classes of sincipital or anterior and occipital or posterior tumours; and, besides anatomical divisions, founded on the particular region, each group is subdivided according to the nature of the contents of the tumour into Meningoceles, Hydrencephaloceles, and Cenencephaloceles; the first containing fluid only, the last brain substance, and the second containing more or less of each. Meningoceles are of least, and cenencephaloceles of most frequent occurrence. Sincipital Encephaloceles of all kinds arise alike from increased intracranial pressure during fœtal life, from delayed absorption or abnormal persistence of that portion of the amniotic membrane that originally covered the fœtal head, or the "hydrops ventriculi saccatus" of Virchow. From the structure of the cerebrum such tumours are necessarily unilateral, except occipital meningoceles, which may be in fact upward extensions of the condition known as Spina bifida. These are not amenable to treatment, nor are some of the larger occipital cenencephaloceles, the so-called "herniæ cerebri." But in all others Bergmann advises early operation, as large masses of even the frontal lobes may be removed without any ill effects. The skin, which is usually highly vascular, he divides by a circular incision, having previously arranged a ligature around the pedicle. The tumour is then taken off by a single stroke of the knife, and the ligature drawn tight. This procedure is most satisfactory in the case of cenencephaloceles, for the existence of a hydrocephalous condition, over which the operation has no influence, not infrequently interferes with the permanency of the result in that of meningoceles.

In the treatment of hydrocephalus he advises puncture, as also in that of meningitis, the so-called acute hydrocephalus; for, though it has not as yet, in his experience, effected a cure, he has seen such relief in a case of basilar meningitis, in which he punctured the lateral ventricle, that, as in the now recognised treatment of the somewhat parallel condition of tubercular peritonitis, it is, he thinks, possible that the restoration of the normal circulation, which follows the removal of the pressure of the effusion, might occasionally lead to more lasting results. He maintains that deep seated abscesses of the brain may, in some instances, be set up by *acute* inflammation, contrary to the generally received opinion. Syphilitic gummata should always be treated by a course of mercury and potassium iodide; but his own experience has satisfied him that, this failing, they are not beyond the scope of surgical operation any more than gliomata and other tumours that have furnished some of the greatest triumphs of modern surgery.

